# High Quality–Factor All–Dielectric Metacavity for Label–Free Biosensing

**DOI:** 10.1002/advs.202410125

**Published:** 2024-11-18

**Authors:** Yuqiao Zheng, Jiacheng Sun, Yaqing Ma, Hongyong Zhang, Zhen Cui, Giannis G. Paschos, Xixi Song, Ying Tao, Pavlos Savvidis, Wei Kong, Liaoyong Wen, Sumin Bian, Mohamad Sawan

**Affiliations:** ^1^ Zhejiang University Hangzhou Zhejiang 310058 China; ^2^ CenBRAIN Neurotech Center of Excellence School of Engineering Westlake University Hangzhou Zhejiang 310030 China; ^3^ Key Laboratory of 3D Micro/Nano Fabrication and Characterization of Zhejiang School of Engineering Westlake University Hangzhou Zhejiang 310030 China; ^4^ Advanced Solid‐state Semiconductor Lab School of Engineering Westlake University Hangzhou Zhejiang 310024 China; ^5^ Key Laboratory for Quantum Materials of Zhejiang Province Department of Physics School of Science Westlake University Hangzhou Zhejiang 310030 China

**Keywords:** biosensor, high quality‐factor, metasurface, microcavity, virus detection

## Abstract

High sensitivity and high quality‐factor are crucial for achieving outstanding sensing performance in photonic biosensors. However, strong optical field confinement and high light–biomolecule interactions on photonic surfaces are usually contradictory and challenging to satisfy simultaneously. Here, a distinctive configuration for addressing this issue is reported: embedding a nanophotonic metasurface inside a micro vertical cavity as a meta‐channel (metacavity) biosensor. The analyte solution serves as the cavity medium, thereby maximizing the light–analyte interaction. Simulation validation is conducted to optimize the metacavity with high structural robustness and remarkable optical and sensing properties. Large‐scale low‐cost metacavity biosensors are realized by combining anodic aluminum oxide template technique and wafer bonding. Experimentally, the metacavity biosensor demonstrates a notable quality‐factor (maximum 4140) and high bulk refractometric sensitivity (450 nm RIU^−1^), resulting in an unprecedented figure‐of‐merit (1670 RIU^−1^). Moreover, the metacavity biosensor achieves high surface sensitivity, together with a detection‐limit of 119 viral copies mL^−1^ for label‐free severe acute respiratory syndrome coronavirus 2 (SARS‐CoV‐2) pseudovirus sensing, revealing remarkable performance in both bulk and surface sensing.

## Graphical abstract

To address the challenge of balancing strong optical field confinement with the associated decrease in sensitivity in high‐quality‐factor photonic resonator sensors, a distinct analyte medium metasurface‐enhanced microcavity (metacavity) biosensor is introduced. Owing to its ingenious design, the metacavity maintains an ultra‐high Q‐factor with a notable sensitivity, leading to an exceptional figure‐of‐merit. The overall performance surpasses that of conventional resonator sensors.

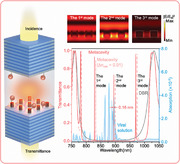

## 1 Introduction

Viral pandemics pose a severe threat to public health, underscoring the critical importance of robust detection methods. Optical biosensors have attracted considerable attention owing to their high sensitivity, versatility, and rapid response in monitoring infectious diseases. They can quantitatively measure the local refractive index (RI) change induced by the biorecognition element–target binding at the sensing surface, after which an advanced analyser transforms the changes in the optical properties into readable response signals (*i.e*., resonance wavelength shift). Therefore, realizing strong optical confinement on sensor surfaces to gain efficient light–biomolecule interactions is a fundamental requirement for optical biosensors. Among various types of optical biosensors, micro‐/nano‐photonic biosensors based on plasmonics or dielectrics are emerging because of their outstanding ability to manipulate light in subwavelength volumes.^[^ ^1^, ^2^, ^3^ ^]^ In photonic resonators, light circulates temporally within the cavity, thereby amplifying light–matter interactions, which dominate the sensitivity of resonators when used as sensors. The resonance properties can be adjusted by tuning the structures or materials of photonic resonator sensors to accommodate various bioanalytes.^[^ ^1^, ^4^ ^]^

Plasmonic resonators have attracted great attention in biosensing because they impose strong localized field confinement within a nanoscale decay length, leading to extremely high surface sensitivity, such as localized surface plasmon^[^ ^5^, ^6^ ^]^ and surface lattice resonance.^[^ ^7^ ^]^ However, the quality (Q)‐factor of plasmonic resonator is limited by the inherent high material Ohmic loss or far‐field radiative loss, usually in the order of tens.^[^ ^1^, ^8^, ^9^, ^10^, ^11^ ^]^ Even if other emerging modes, such as bound stated in continuum (BIC),^[^ ^12^, ^13^, ^14^ ^]^ Fabry–Pérot,^[^ ^15^ ^]^ or Fano resonance^[^ ^16^ ^]^ have been proposed for sensing applications, the enhanced Q‐factor can only reach the order of hundreds.

Dielectric photonic resonators based on different mechanics have been proposed to address the limitations of metal materials as sensing components, such as nanoscale BIC metasurfaces,^[^ ^17^, ^18^, ^19^, ^20^, ^21^, ^22^, ^23^ ^]^ waveguides,^[^ ^24^ ^]^ microscale spheres,^[^ ^25^, ^26^ ^]^ bubbles,^[^ ^27^ ^]^ disks,^[^ ^28^ ^]^ and toroids^[^ ^29^ ^]^ They offer competitive near‐field enhancement at nanoscale volume with minimum material loss. So far, high Q‐factors were reported for dielectric quasi‐BIC metasurface sensors,^[^ ^17^, ^18^, ^19^ ^]^ and an ultrahigh Q‐factor exceeding 10^6^ was achieved for high‐order Mie resonance–based whispering gallery sensors.^[^ ^27^ ^]^ However, a tightly confined light field in a high‐Q resonator inevitably loses its interaction with the ambient environment, leading to low resonance sensitivity. Such trade‐off has become a major challenge for photonic resonator sensors.^[^ ^23^, ^30^, ^31^, ^32^ ^]^ As a result, further enhancement of the optical sensing performance is constrained by the trade‐off between high Q‐factor and high resonance sensitivity.

In this study, we address this inherent contradiction by proposing an all‐dielectric metacavity biosensor with nanoscale silicon nitride (Si_3_N_4_) metasurface embedding inside a distributed Bragg reflector (DBR) microcavity for label‐free intracavity biosensing. A distinct configuration was proposed by having the analyte solution directly serves as the cavity medium to maximize light–analyte interactions. Simulation results proved that the metacavity achieves extreme localized field enhancement at the material–environment interface, with the field tail penetrating the solution. Moreover, the metacavity exhibits an extreme Q‐factor as a typical dielectric photonic resonator, while delivering high sensitivity as a surface plasmonic resonator. Consequently, an unprecedented figure‐of‐merit (FoM) is obtained. Experimentally, large‐scale metasurfaces were fabricated on DBRs based on anodic aluminum oxide (AAO) template technique. Microcavity devices were then fabricated by Cu─Cu bonding of two DBR wafers with precisely controlled cavity parameters. The Si_3_N_4_ metacavity, with customized structural parameters, exhibited an average and maximum Q‐factor of 3706 and 4140, respectively, with a sensitivity of 449.43 nm RIU^−1^ and an FoM reaching 1669 RIU^−1^ in bulk refractometric sensing. The comprehensive sensing performance experimentally surpassed that of any previously reported photonic resonator sensors, highlighting the reliability of our metacavity as a viable solution for balancing both high Q‐factor and high resonance sensitivity. Furthermore, the metacavity exhibited remarkable surface sensitivity, which was over three times greater than that of a bare microcavity. It also achieved an outstanding limit of detection (LoD) of 119.51 viral copies mL^−1^ for label‐free severe acute respiratory syndrome coronavirus 2 (SARS‐CoV‐2) pseudovirus detection. In summary, the all‐dielectric metacavity configuration presents significant benefits, enabling the attainment of superior optical characteristics alongside impressive sensing capabilities, rendering it a competitive solution for next generation biosensing platform.

## 2 Results

### 2.1 Metacavity Design and Optimization

**Figure 1:**
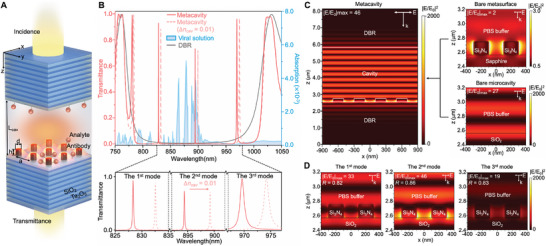
Illustration of the all‐dielectric analyte medium metacavity. (A) Schematic of the metacavity. A normal plane wave was coupled to the DBR microcavity (characterized by cavity length L_cav_). The square lattice dielectric metasurface (characterized by height h, diameter d, and inter‐atom distance a) was embedded at the inner surface of the bottom DBR. Sample solutions were injected into the cavity to directly serve as cavity media. Viral particles selectively adhered to the biorecognition element—a capture antibody immobilized on the silica surface via a silica‐binding protein. (B) Simulated transmission spectra of the bare DBR (grey line), the optimized metacavity ((L_cav_ = 3000 nm, 10 pair DBR, a = 400 nm, h = 180 nm, d = 200 nm); red line), and the optimized metacavity with intracavity RI increased by 0.01 (red‐dashed line). Experimental absorption spectrum of the SARS‐CoV‐2 pseudovirus solution is presented by the blue area. Detailed views of the three modes are displayed at the bottom part. (C) Left: electric field distribution of the 2nd mode of the optimized metacavity. Right (top): electric field distribution of the bare metasurface at the working wavelength. Right (bottom): electric field distribution of the 2nd mode of the bare microcavity. (D) Electric field distribution of the 1st (left), 2nd (middle), and 3rd (right) mode of the optimized metacavity.

The concept diagram of the analyte medium metacavity biosensor is presented in **Figure** **1**A. Such “metacavity” configuration, in which metasurfaces are coupled within planar microcavities, presents a promising solution for enhancing the 3D light manipulation capability.^[^ ^33^, ^34^, ^35^ ^]^ The combined mode offers high tunability by adjusting the structural parameters and materials^[^ ^36^, ^37^ ^]^ of both the DBR microcavity and the metasurface.

Herein, a DBR containing 10 pairs of alternating Ta_2_O_5_ (107 nm)/SiO_2_ (150 nm) films was used, with a stopband between ≈770 and ≈1020 nm (Figure 1B: grey line). This spectral range was designed with respect to the absorption properties of the viral particle solutions (Figure 1B: blue area). A 2D square lattice Si_3_N_4_ nanocylinder metasurface characterized by its period (a), diameter (d), and height (h), was embedded at the bottom DBR surface (Figure 1A). The biointerface was constructed on the inner surface of the microcavity. When the metacavity medium was set to be phosphate buffered saline (PBS) buffer, three resonance modes with high‐Q‐factor and large free spectral range (FSR) were obtained: the 1st, 2nd, and 3rd modes at 828 , 894 , and 969 nm, respectively. The simulated transmittance spectra for these modes are illustrated in Figure 1B (red line). Among them, the 2nd mode exhibited a superior resonance Q‐factor of 5320 and an exceptional full width at half maximum (FWHM) at 0.16 nm. Such high Q‐factor confers considerable robustness to fluctuations in structural parameters (Figure S1A, Supporting Information), attributing to the low absorption, and high intrinsic material Q‐factor (10^3^–10^4^) and dilution factor (10^6^) of Si_3_N_4_.^[^ ^38^ ^]^ Moreover, when we adjust the L_cav_ of a bare microcavity so that its resonance mode aligned with that of the metacavity at the 2nd mode, the metacavity still provides an 8% enhancement in the Q‐factor (Figure S1B, Supporting Information). Although dielectric sensors typically have a high Q‐factor, they often demonstrate diminished sensitivity to environmental RI variations due to limited light field overlap with the surroundings compared with surface plasmonic sensors. Nevertheless, the metacavity, owing to its ingenious design, has transcended this limitation. It not only maintains an exceptionally high Q‐factor, but also achieves a notable bulk sensitivity of 483 nm RIU^−1^ at the 2nd mode across the 1.33–1.35 RI range. Consequently, the metacavity attains a superior FoM*_Bulk_* of 2877 RIU^−1^. The results highlight that our metacavity delivers both the ultra‐high Q‐factor and FoM typically associated with dielectric sensors, and the exceptional sensitivity characteristic of plasmonic sensors, resulting in unparalleled in traditional photonic resonator sensors.

Furthermore, the combination of microcavity and intracavity metasurface yields an exceptionally localized light field, resulting in unprecedented high mode overlap factor (R‐factor). At the 2nd mode, strong light confinement was achieved at the material–environment interface, with the field tail penetrating the analyte medium (Figure 1C), thereby significantly amplifies the light‐analyte interaction. Moreover, such high near‐field enhancement factor (EF) demonstrates exceptional stability, remaining consistent despite changes in device parameters (Figure S1C, Supporting Information). The superior properties can be ascribed to the coupling of the bare metasurface mode in the x–y plane (Figure 1C, right top) with the bare microcavity mode along the z direction (Figure 1C, right bottom) at the 2nd mode. Consequently, a higher R‐factor was gained for the 2nd mode (0.86) compared to that of the 1st (0.82) and 3rd (0.83) modes (Figure 1D). The analytical definition of R‐factor for our intracavity sensor was provided in the Experimental Section. These properties underscore the 2nd mode's distinct advantage in sensing applications, empowering metacavity a crucial candidate for ensuring the combination of high optical performance and exceptional sensing capabilities.

**Figure 2:**
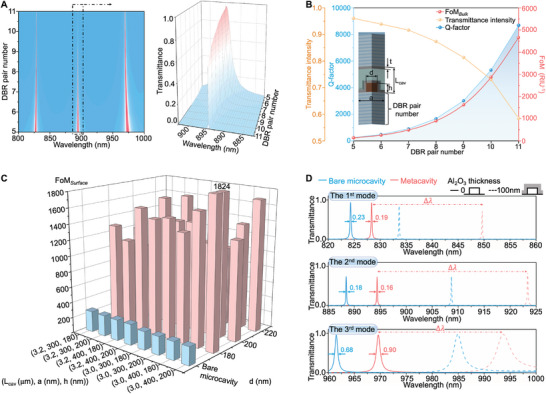
Sensing capabilities of the microcavity and metacavity sensors. (A) Left: Variation of transmittance spectra with respect to DBR pair number change from 5 to 11. Right: Zoom in view of the 2nd mode. (B) Variation of the Q‐factor (blue region), the transmittance intensity (yellow), and FoM*_Bulk_* (red) with respect to DBR pair number change from 5 to 11. The key parameters of metacavity, together with the illustration of the adsorbate layer (thickness: t) deposited inside the metacavity sensing surface are presented on the left side of the chart. (C) Simulated surface FoM variations of the microcavity sensors with respect to structural parameters (L_cav_, a, h, and d) when surface adsorbate layer was varied from 0 to 120 nm. (D) Resonance wavelength shift of the 1st (top), 2nd (middle), and 3rd (bottom) modes of the optimized metacavity (red lines) and bare microcavity (blue lines) with a 100‐nm Al_2_O_3_ thin film deposited on the sensing surface.

The DBR designs exert a pivotal influence on the sensing capabilities of the metacavity, and should be precisely engineered. Intuitively, with the increment of DBR pair number from 5 to 11, sharper resonance with higher Q‐factor and FoM*_Bulk_* was observed for the 2nd mode (**Figure** **2**A,B). Considering the continuously decreasing transmittance intensity, a 10‐pair DBR optimally combines high optical properties and exceptional bulk sensing performance. Regarding surface sensing, the intensity of light‐analyte interactions within the immediate proximity of the sensing surface is the decisive factor. To quantitively investigate the surface sensing properties, different thicknesses of adsorbate layers (Al_2_O_3_) were added to the inner surface, based on which we calculated the surface sensitivity factor (*m*) and FoM*_Surface_*. The analytical framework of *m* is described in detail in the Experimetal Section. To facilitate a comparable assessment against bulk sensitivity, the FoM*_Surface_* is succinctly defined as the ratio of *m* to the FWHM, as *m* is comparable to the bulk sensitivity.^[^ ^13^, ^39^ ^]^ Overall, metacavities consistently demonstrate superior surface sensing performances compared with bare DBR microcavities. When tuning the structural parameters, approximately four‐times enhancements in *m* (Figure S2, Supporting Information) and eight‐times enhancements in FoM*_Surface_* (Figure 2C) were obtained for the metacavities compared to the bare microcavities. It is noteworthy that for a metacavity, under suboptimal structural designs, the 2nd mode exhibits a higher near‐field EF and Q‐factor, but does not yield higher R‐factor, as exemplified in Figure S3 (Supporting Information). The relative sensitivities of the 2nd mode are therefore diminished compared with the other modes (Figure S4, Supporting Information). Considering this, for the optimized metacavity in Figure 1 (L_cav_ = 3000 nm, 10 pair DBR, a = 400 nm, h = 180 nm, d = 200 nm), while a 100‐nm Al_2_O_3_ thin film was added to mimic the surface‐binding viral particles, a clearer spectrum shift was observed (Figure 2D: red lines) compared with that of the bare DBR microcavity (Figure 2D: blue lines). Meanwhile, corroborating our earlier analysis, the 2nd mode evidently provides the highest response signal level among the three modes for the metacavity. These comparisons underscore the optimized metacavity's enhanced sensing performances and establish its advantage for sensing applications.

Overall, the proposed all‐dielectric metacavity configuration, in theory, confers crucial advantages for sensing applications. It ensures a higher Q‐factor, together with strong light confinement in the medium environment close to the sensing surface, thereby enhancing light‐matter interactions within the sensing volume. As a result, the meticulously optimized metacavity demonstrates equivalent sensitivity to plasmonic sensors, while simultaneously possessing an exceptionally high Q‐factor and FoM. These properties firmly establish the metacavity as a strong candidate for ensuring the integration of superior optical properties and exceptional sensing capabilities.

### 2.2 Metacavity Sensor Fabrication and Characterization

**Figure 3:**
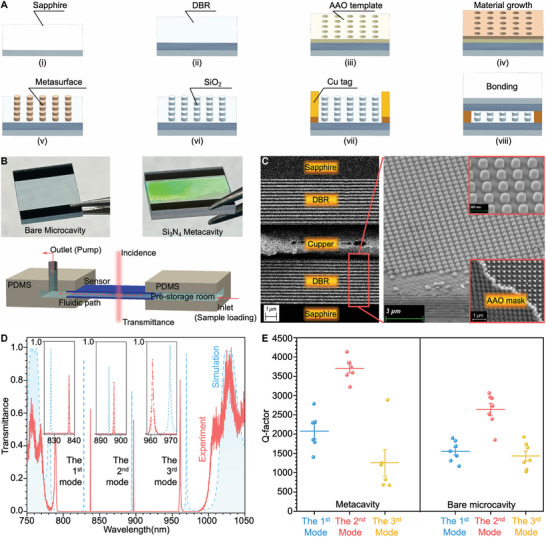
Fabrication and characterization of the metacavity and bare microcavity sensors. (A) Overview of the metacavity fabrication steps: (i, ii) DBR deposition on a sapphire substrate; (iii) transfer of a large‐scale AAO template; (iv) formation of the metasurface and subsequent (v) mask removal; (vi) SiO_2_ layer deposition; (vii) Cu tag deposition; and (viii) Cu─Cu bonding. (B) Top: photographs of the bare microcavity (left) and metacavity (right). Bottom: schematic of the microfluidic channel for sample injection. (C) SEM images of the cross section of the cavity (left) and the metasurface (right and top inset image), alongside the AAO template (bottom inset image). (D) Simulation (blue) and experimental (red) normalized transmission spectra of the optimized metacavity and a zoom‐in of the three modes. (E) Experimental Q‐factor variations of the three modes of the optimized metacavity and bare microcavity sensors.

To ensure mass producible metacavity sensors with consistent performance and precisely controlled cavity parameters, we utilized the AAO template technique for crafting large‐scale metasurfaces, and material bonding for the precise, wafer‐scale creation of fixed‐length cavities. The detailed process steps involved in metacavity fabrication are presented in **Figure** **3**A. First, Ta_2_O_5_/SiO_2_ DBR was deposited on a sapphire substrate. Then, the AAO template was transferred onto the DBR, followed by Si_3_N_4_ growth and template removal via N_2_ flow. The fabrication details of the AAO template have been reported in our previous works.^[^ ^13^, ^40^, ^41^ ^]^ For the metacavity, an additional 5‐nm SiO_2_ thin film was deposited on the Si_3_N_4_ metasurface to enable biofunctionalization. Cu tags were then deposited on both sides of the DBR chips, and the two chips were bonded together to form the metacavity, where a consistent 220‐kg pressure was applied at 350 °C for 30 min within a vacuum chamber (Figure S5A, Supporting Information). This process facilitated the formation of new Cu─Cu metallic bonds at the material interface, thus ensuring a well‐controlled L_cav_ determined by the thickness of the Cu tags. A similar process was adopted for the fabrication of bare microcavities, in which two bare DBR chips were bonded together, with steps (i), (ii), (vii), and (viii) involved. Figure 3B shows photographs of the bare microcavity (left) and the metacavity (right). Finally, the sensor was enclosed within a preprepared polydimethylsiloxane (PDMS) template to form a microfluidic system (Figure 3B, bottom). Via the microfluidic channel, the liquid was directed into a prestorage room, injected into the microcavity, and exited from the opposite side of the sensor. Experiments in this study were carried out using PBS buffer (1 ×) as a cavity medium unless otherwise stated. Figure S5B (Supporting Information) presents a real‐sample image of the microfluidic system, highlighting the liquid‐filling process within the cavity. Regulating the fluid filling speed according to the fabrication conditions and sensor design is crucial because the flow introduces additional pressure and alters the cavity length. The microfluidic speed was maintained at 1 µL min^−1^ in this work. Figure 3C shows the cross‐sectional scanning electron microscopy (SEM) images of the microcavity, including the Cu─Cu interface after bonding (left) and the DBR wafer with the Si_3_N_4_ metasurface (right). The two detailed views on the right side depict the metasurface (top) and the partially removed AAO template (bottom). The energy dispersive spectroscopy of the metasurface functionalized DBR is also presented in Figure S5C (Supporting Information).

Owing to the meticulously designed fabrication techniques, we have experimentally realized extreme Q‐factors that demonstrate significant robustness. Generally, the experimental transmittance spectrum corresponds with the simulated result (Figure 3D). Consistent with theoretical predictions, the optimized metacavity exhibits higher Q‐factor (3706 ± 290, maximum 4140) compared with the bare microcavity (2638 ± 422, maximum 3065). This enhancement arises from the defects in real clinical samples, such as the imperfect parallelism of the DBRs, and the scattering caused by the rough inner surface, which lead to light field leakage in both the x‐ and y‐directions. The high‐index metasurface enhances the confinement of the optical field within the x‐y plane, thereby increasing the Q‐factor. Moreover, the standard deviations (SD) of both the Q‐factor (Figure 3E) and the FWHM (Figure S5D, Supporting Information) were significantly lower for the 2nd mode compared to the 1st and 3rd modes. This result demonstrates the better confinement of the 2nd mode in to the microcavity compared to the 1st and 3rd modes. Therefore, we designated the performance of the 2nd mode as the sensor capability benchmark in subsequent tasks.

### 2.3 Sensing Capacity of the Metacavity and Microcavity Sensors

Subsequently, we carried out sensing experiments to substantiate our theoretical analysis. The experiments were divided into three main parts: evaluating the bulk sensing performance, constructing the surface sensing model, and verifying whole virus biosensing capabilities.

**Figure 4:**
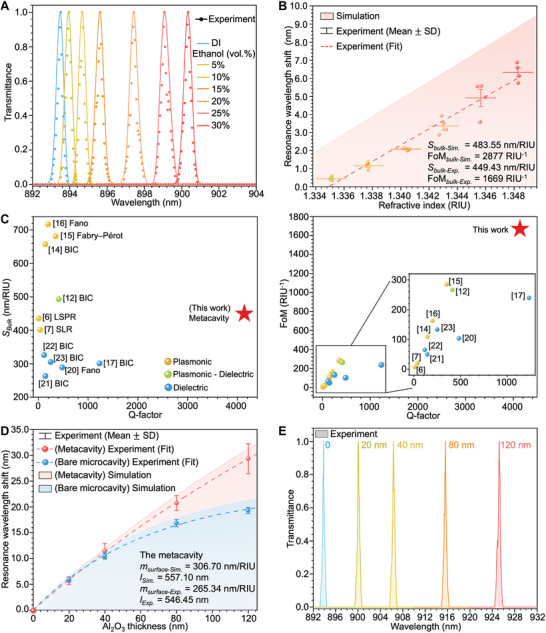
Sensing capabilities of the metacavity and bare microcavity sensors. (A) Normalized transmittance spectra of one of the metacavities with cavity been filled with DI and different concentrations of aqueous ethanol solutions (5–30 vol%) sequentially. (B) Simulation and experimental resonance wavelength shift results of the metacavity with RI change. (C) Experimental Q‐factor, *S_Bulk_* (left), and FoM*_Bulk_* (right) of recent photonic resonator refractometric sensors. (D) Simulation and experimental resonance wavelength shift results of the bare microcavity (blue) and metacavity (red) as a function of Al_2_O_3_ thin film thickness. (E) Transmittance spectra of one of the metacavities with inner surface deposited with different thicknesses of Al_2_O_3_ thin film.

The bulk refractometric sensing was conducted by adjusting the ethanol volume ratios in deionized water (DI) from 5% to 30% as intracavity medium. The associated RI values are listed in Table S1 (Supporting Information). Evidently, clear resonance wavelength shifts were observed with the increment in RI, with **Figure** **4**A presents the normalized resonance shifts of one of the samples. The complete spectral data are detailed in Figure S6A (Supporting Information). Overall, by exploiting the potent light‐confining properties of the metacavity, we achieved an ultrahigh experimental FoM (1669.34 ± 237.51 RIU^−1^) with a satisfactory S*_Bulk_* (449.43 ± 64.33 nm RIU^−1^), which aligned well with the simulation results as illustrated in Figure 4B. Further analysis of recent photonic resonator sensors, in terms of experimental Q‐factor, S*_Bulk_*, and FoM*_Bulk_*, confirms the metacavity's comprehensive superior performance, as illustrated in Figure 4C. The pertinent details of the referenced works are concisely encapsulated in Table S2 (Supporting Information). Benefitting from its high Q‐factor and S*_Bulk_*, the metacavity achieves an unprecedented high FoM*_Bulk_*, outperforming most currently reported plasmonic and dielectric resonators. Furthermore, the detection range of a resonator is largely dependent on the FSR. Owing to the large FSR (simulation: 75 nm), the metacavity can offer high bulk sensing performance across a broad RI range typically encountered in bioanalytes (1.33–1.49) as illustrated in Figure S6B (Supporting Information). This distinct advantage situates the metacavity as a promising solution for achieving an optimal balance between high optical and sensing performance.

The second part focuses on the surface sensing model construction, which affirms the metacavity's enhanced surface sensing capabilities over that of a bare microcavity. To mimic the label‐free biosensing toward the monoclonal antibody (mAb) and whole virus (dimension scales: ≈10 nm and ≈100 nm, respectively), Al_2_O_3_ thin films of various thicknesses (20, 40, 80, 120 nm) were grown across different regions of the DBR wafer. In addition to sensitivity, the decay length (*l*) is calculated to represent the rate at which surface sensitivity diminishes, as defined in the Experimental Section. For bare microcavity, a modest sensitivity factor (m*_Exp_*_._ = 80.82 nm RIU^−1^, m*_Sim._* = 87.25 nm RIU^−1^) and decay length (*l_Exp_*_._ = 132.13 nm, *l*_Sim._ = 131.84 nm) were obtained, as shown in Figure 4D. In comparison, the metacavity owns significant spectral response to the increasing Al_2_O_3_ film thickness (Figure 4E), with exceptional experimental sensitivity factor (*m_Exp._* = 265.34 nm RIU^−1^) and decay length (*l_Exp_*_._ = 546.45 nm) that well matched the simulation results (*m_Sim_*_._ = 306.70 nm RIU^−1^, *l_Sim._* = 557.10 nm). The above evidence affirms metacavity as a superior choice for high surface sensitivity biosensors.

### 2.4 Biosensing Capacity of the Metacavity and Microcavity Sensors by SARS‐CoV‐2 Pseudovirus

In an era of deep globalization, pathogens can swiftly spread across the globe, leading to continuous public exposure to evolving viruses and inflicting significant damage on public health and the global economy. This underscores the critical importance of label‐free sensing device for direct whole virus detection, which does not require extra procedures like nucleic acid amplification or virus lysis. Therefore, for the third part, we validate the metacavity's capability in whole virus sensing. Here, first, an oriented and efficacious biofunctionalization strategy is essential to ensure enhanced sensitivity. Biofunctionalization of dielectric devices often involves the use of toxic reagents such as piranha for surface treatment.^[^ ^42^ ^]^ This is particularly the case when a precise orientation of biorecognition element binding is desired to enhance sensitivity. Moreover, classical biofunctionalization often requires other hydrophilic treatments, such as plasma cleaning, which can be impractical for well‐packaged sensor chips. Therefore, in this study, a label‐free biosensing approach was employed using a recombinant protein bioengineered in‐house to directly link the target mAb onto the sensor surface. This recombinant protein, referred to as silica‐binding protein with a core functional domain (cSP),^[^ ^43^ ^]^ has recently demonstrated its efficacy in precisely and conveniently immobilizing mAb onto silica surfaces in a site‐oriented manner, facilitating accurate binding with the target SARS‐CoV‐2 Omicron BF.7 pseudovirus—a prevalent Omicron sublineage during the study period.

Next, before whole virus sensing, the binding efficiencies of cSP and antibody on the metacavity sensor should be first validated, following the process flow in Figure S7A (Supporting Information). This work adopted mAb bebtelovimab, a human antibody with broad neutralizing activity toward Omicron subvariant.^[^ ^44^, ^45^ ^]^ As a result, the spectral response signal for cSP functionalization was less distinct, with an average signal intensity below three times the SD of the control (blank) groups (Figure S7B,C, Supporting Information). Such discrepancy is expected, considering the cSP protein's size of ≈2 nm, which exceeds the detection limit of our sensing system. This suggests that our sensing platform requires refinement to boost its capability in small molecule detection. On the other hand, successful biofunctionalization was confirmed by the clear response signals observed during antibody binding.

**Figure 5:**
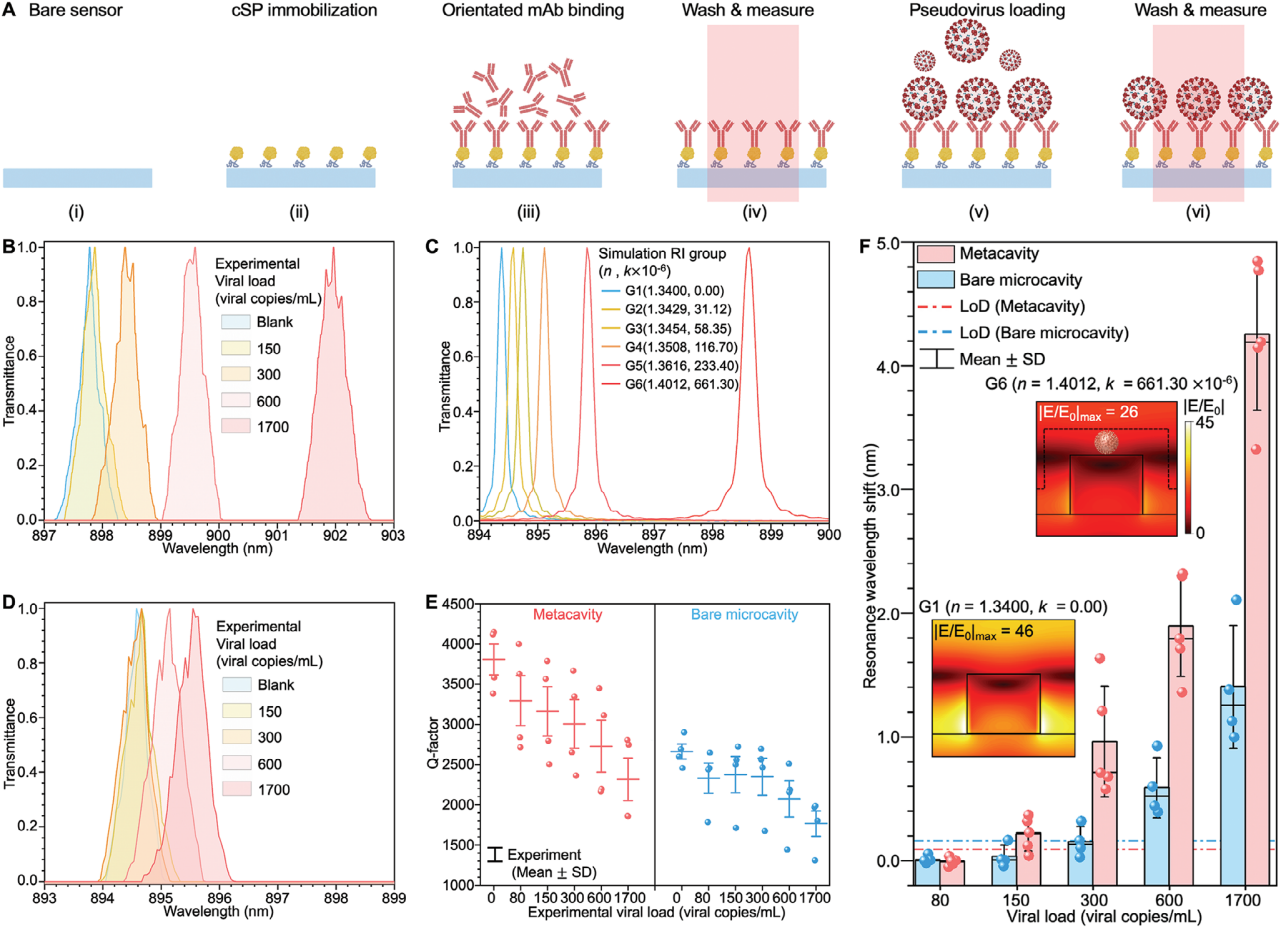
Sensing capabilities of the microcavity and metacavity sensors. (A) Biosensing procedure: (i and ii) cSP protein binding on the bare sensor surface; (iii) oriented functionalization of mAb on the sensing surface; (iv) sensor washing and spectra measurement; (v) pseudovirus sample loading; and (vi) sensor washing and spectra measurement. (B) Normalized experimental transmittance spectra of one of the metacavity sensors in response to different virus concentrations. (C) Normalized simulation transmittance spectra as a function of RI (*n*, *k*) change. (D) Normalized experimental transmittance spectra of one of the bare microcavity sensors in response to different virus concentrations. (E) Experimental Q‐factor changes in the metacavity (red) and bare microcavity (blue) sensors in response to different virus concentrations. (F) Experimental resonance wavelength shifts in the metacavity (red) and bare microcavity (blue) in response to different virus concentrations. The electric field distribution change with the variation in simulated RI values is also presented.

Consequently, we validated the biosensing capabilities of the metacavity sensor both theoretically and experimentally using the SARS‐CoV‐2 BF.7 pseudovirus at varying viral loads (0, 80, 150, 300, 600, and 1700 viral copies mL^−1^). Here, the numerical investigations were conducted based on a set of RI (*n*, *k*) values (Table S3, Supporting Information) established according to the measured (*n*, *k*) values of the viral solutions (Figure S7D, Supporting Information). Regarding experiment, the sequential sample loading procedure (**Figure** **5**A) involves (i) buffer infiltration; (ii) binding of cSP; (iii) site‐orientated immobilization of the capture antibody; (iv) washing and spectrum measurement (the results are saved as the signal for blank group); (v) sample loading; and (iv) washing and spectrum measurement. As a result, the metacavity offers considerable resonance shift scale even under lower viral concentrations. The experimental resonance shift behaviors (Figure 5B) were consistent with the simulation results (Figure 5C). In comparison, the bare microcavity exhibited a significantly lower response signal level than the metacavity, as illustrated in Figure 5D. This confirms the metacavity's superior surface sensitivity as the proposed surface sensing model.

On the other aspect, when the sensor's working wavelength matches the absorption band of the target analyte solution, a variety of sensing signal forms can be used. Accordingly, a significant decrease in the Q‐factor was observed as virus concentration rises. This degradation trend was monitored for both sensors, with a more pronounced effect observed in the metacavity sensor, as illustrated in both simulation (Figure S7D, Supporting Information) and experiment (Figure 5E) results. The observed Q‐factor degeneration is primarily due to the absorption effects caused by viral particles. This suggests that the metacavity confers superior sensitivity to absorptive media, positioning it as an ideal candidate for the detection of inherently absorbent analytes.

Beyond evaluating response sensitivity, it is imperative to quantify the metacavity's LoD for viral detection, determined in accordance with the formula detailed in the Experimental Section. Consistent with the surface sensing model mentioned earlier, the metacavity showed a markedly superior response signal level when detecting viral‐scale markers compared to the bare microcavity (Figure 5F). As a result, the LoD of the metacavity was 119.51 viral copies mL^−1^, which was significantly better than that of the bare microcavity (306.29 viral copies mL^−1^). Moreover, under different RI (*n*, *k*) values, a clear near electric field distribution change with near‐twofold decline in the electric field magnitude can be observed (Figure 5F). The reduction in the near‐field EF further validates the efficacy of metacavity in virus detection, particularly in leveraging a variety of response signal patterns. Such performance is highly competitive compared to commercial SARS‐CoV‐2 detection kits, which typically have LoD levels of ≈80–150 viral copies mL^−1^,^[^ ^46^ ^]^ while also surpassing most recent studies in whole virus detection (Table S4, Supporting Information). Furthermore, the metacavity is inherently well‐sealed, ensuring that the detection region is internal to the cavity and shielded from the external environment. The less dependent of metacavity on bulky and costly equipment (e.g., electrochemical workstation or Raman spectrometer) positions it as a strong contender for practical applications demanding high sensitivity.

## 3 Conclusion

We have demonstrated a novel “analyte medium metacavity biosensor” configuration. A nanoscale metasurface was embedded inside a DBR microcavity for 3D light manipulation, and the analyte solution acts directly as the cavity medium to maximize light–analyte interactions. Two sensor configurations were evaluated: Si_3_N_4_ metacavity and bare microcavity. Simulations and experiments were conducted for bulk refractometric sensing and surface sensing capability analysis. Numerical results proved that the metacavity exhibits strong localized field confinement at the material–environment interface, with the field tail penetrating the analyte solution while maintaining a high Q‐factor. The AAO template technique and wafer bonding strategies were adopted for large‐scale and mass‐production devices with the potential to fabricate wafer‐scale devices. Both sensor configurations presented notable optical and sensing properties, with the optimized metacavity having an extreme and robust experimental Q‐factor (3706 ± 290, maximum 4140) and bulk refractometric sensing FoM (1669 RIU^−1^) while maintaining a considerable sensitivity (449.43 nm RIU^-1^). The comprehensive performances surpass those of previously reported resonator‐based biosensors. Surface sensitivity was also estimated by theoretical model construction and whole virus biosensing based on the SARS‐CoV‐2 pseudovirus. As a result, the metacavity provides outstanding surface sensitivity and LoD (119.51 viral copies mL^−1^) in label‐free SARS‐CoV‐2 pseudovirus detection.

There remain factors to be improved and investigated in future work. First, from the perspective of sensor design, engineered intracavity microfluidic channels are preferred to maximize the mode overlap integral of the dominant mode, thereby exploring the sensing performance to its maximal. Second, the metacavity devices require a minimum sample volume of 0.18 µL for full filling. However, owing to limitations of the microfluidic system, each detection in this work necessitates 50 µL, resulting in significant wastage. Future work should prioritize an improved microfluidic system to minimize sample waste. Additionally, the reliance of high Q‐factor devices on high‐resolution spectrometers restricts their applications in portable and point‐of‐care diagnostics. However, recent advances have introduced imaging‐based spectrometer‐free biosensing platforms that are well‐suited for cost‐effective, high‐Q sensing applications using all‐dielectric materials.^[^ ^21^, ^23^ ^]^ For instance, by monitoring the spectral intensity under single‐wavelength incidence, the high‐Q photonic BIC resonator enables the detection of ultrasmall RI changes.^[^ ^23^ ^]^ Furthermore, the increasing prevalence of coinfection by multiple viruses and new pandemics (*e.g*., Disease X^[^ ^47^ ^]^), along with more severe symptoms,^[^ ^48^ ^]^ highlights the significance of multivirus detection.^[^ ^4^, ^49^ ^]^ Customized metacavity designs are required to achieve optimal sensing performance for various viruses; however, the cost of conventional numerical methods is high. Therefore, employing advanced deep learning methods to customize metacavity designs tailored to different viruses is a promising approach to achieve optimal sensing performance. One step forward from the perspective of sensor applications, metacavity chips have the advantage of creating isolated volumes for single‐layer cell and organoid culture. These characteristics make them potentially ideal containers for organoids, forming a metacavity‐based organoids‐on‐chip platform that has emerged as a cutting‐edge biosystem for disease modeling and personalized medicine.^[^ ^50^, ^51^, ^52^ ^]^ By exploiting imaging techniques, a metacavity‐based organoids‐on‐chip platform has high potential for real‐time and dynamic monitoring and the capability to investigate multiorgan interactions.

## 4 Experimental Section

### Metacavity Design

The spectra and optical fields of the microcavities were calculated by commercial software Lumerical FDTD. The simulations employed an anti‐symmetric condition along x direction, a symmetric condition along y direction, and a perfectly matched layer along the z direction. The mesh precision was set to 10 nm inside the cavity along the x, y, and z directions. The RI values of the Ta_2_O_5_, sapphire, and PBS buffer (1 ×) were measured using an ellipsometer as presented in Figure S8A–C (Supporting Information). The absorption spectrum of the SARS‐CoV‐2 pseudovirus solution was measured using a spectrophotometer with a viral load of 10^4^ copies mL^−1^. The RI value of the SARS‐CoV‐2 pseudovirus solution was measured using an ellipsometer with a viral load of 5000 viral copies mL^−1^ as presented in Figure S8D (Supporting Information). The resolution of the simulated spectrum for the Q‐factor calculation is the same as that of the experimental spectrum.

Regarding sensing properties, according to Yu et al.,^[^ ^27^ ^]^ the relative surface sensitivity (Δλ/λ) is dependent on the electric field magnitude *E*, total energy *U*, vacuum permittivity ε_0_, cavity medium RI *n_medium_*, biomolecule surface mass density *m_Surface_*, and analyte concentration *VL_analyte_*:

(1) $$\frac{\Delta \lambda }{\lambda }=\left(\int \;\;\;\int \varepsilon_{0}{\left|E\right|}^{2}\frac{dS}{2U}\right)n_{medium}m_{surface}\frac{dn_{medium}}{dVL_{analyte}}$$

Therefore, a higher near‐field EF with a smaller mode volume, and larger analyte–hot spot spatial overlap is desired. To investigate this overlapping efficiency effect, Zhou et al. described the R‐factor as the proportion of the electric field within the sensing volume to the entire field intensity.^[^ ^53^ ^]^ For the metacavity proposed in this work, the R‐factor is defined as the ratio of the electric field in the sensing volume to that in the entire region as follows:

(2) $$R=\frac{\int_{z_{cav0}}^{z_{cav0}+L_{cav0}}\langle {\left|E/E_{0}\right|}^{2}\rangle dz}{\int_{z_{0}}^{z_{0}+L}\langle {\left|E/E_{0}\right|}^{2}\rangle dz}$$

where *z*_0_ to *z*_0_  +  *L* denotes the entire region of the device and  *z*_*cav*0_ to *z*_*cav*0_  +  *L_cav_* represents the space inside the cavity.

### Reagents and Facilities

The sapphire wafer and Ta_2_O_5_ and SiO_2_ particles were purchased from ZhongNuo Advanced Material Technology Co., Ltd. (Beijing, China). The supercontinuum laser was purchased from YSL Photonics Co., Ltd. (SC‐5, Wuhan, China). The capture antibody Research Grade Bebtelovimab‐DVV00319 was purchased from AtaGenix (Wuhan, China), and the SARS‐CoV‐2 B.1.1.529 sublineage BF.7 (Omicron) Spike Pseudovirus (PSV‐026) was obtained from Sino Biological (Beijing, China). cSP protein was developed in‐house and bioengineered. All the biomolecules in this study were diluted using fresh PBS buffer (pH 7.4), unless otherwise stated. Ta_2_O_5_ and SiO_2_ films were fabricated via electron beam deposition, whereas Si_3_N_4_ films were fabricated via plasma‐enhanced chemical vapor deposition. The biosafety level‐2 laboratory at Westlake University enabled us to conduct experiments involving the SARS‐CoV‐2 pseudovirus.

### Biosensing Procedures

Initially, the microcavity was filled with a PBS buffer. Subsequently, cSP protein (10 µg mL^−1^) followed by the capture antibody bebtelovimab (20 µg mL^−1^) was injected into the cavity for 20 min. Then, viral solutions were injected into the cavity stepwise, starting from low to high concentrations (referred to as the sample loading process). Each sample loading process took 30 min. Between each concentration of the sample, the cavity was cleaned with PBS buffer for 15 min, after which the transmission spectrum was recorded every minute. During the sample loading phase, when the cavity was filled with the sample solution, spectrum recording was suspended.

Five measurements were taken for each blank group, and three measurements were taken for each viral concentration group. The resonance wavelength shift of each viral concentration point was calculated by averaging the measurements. Four devices were used to estimate the sensing capability of each microcavity sensor. For sensing performance evaluation, the bulk refractometric sensitivity *S_bulk_*, surface sensing resonance wavelength shift,^[^ ^54^ ^]^ FoM, and LoD wavelength were used, which, respectively, defined as follows:

(3) $$S_{bulk}=\Delta \lambda /\Delta n$$

(4) $$\Delta \lambda =m\cdot \left(n_{adsorbate}-n_{medium}\right)\cdot \left(1-e^{-\frac{2t}{l}}\right)$$

(5) $$\mathrm{FoM}\;=\frac{S}{\mathrm{FWHM}}$$

(6) $$\mathrm{LoDw}\mathrm{avel}\mathrm{ength}=\bar{\Delta \lambda_{\mathrm{blank}}}+3\sigma_{\mathrm{blank}}$$

where Δλ represents the resonance wavelength shift, *n_adsorbate_* and *n_medium_* are the refractive indices of the adsorbate and sensing media, respectively. For Al_2_O_3_ thin films and the PBS buffer, *n_adsorbate_* and *n_medium_* can be treated as 1.63 and 1.34, respectively. *l* is the decay length, and σ_*blank*_ is the SD of the blank group. The LoD is determinded through the linear interpolation of LoD wavelength between the two adjacent virus concentration groups.^[^ ^55^ ^]^

### Statistical Analysis

The figures in this study were constructed using OriginPro 2019. The experimental transmittance spectra are normalized. Key data are presented as mean ± SD. The experimental spectra in the sensing sections are presented by experimental data and fitted curves. Each bulk refractometric sensing and biosensing group contains at least four independent sensors.

## Conflict of Interest

The authors declare no conflict of interest.

## Supporting information



Supporting Information

## Data Availability

The data that support the findings of this study are available from the corresponding author upon reasonable request.
