# Supplementary material for: High Quality–Factor All–Dielectric Metacavity for Label–Free Biosensing
Source: Adv Sci (Weinh). 2024 Nov 18;12(4):2410125. doi: 10.1002/advs.202410125 (PMC11775555; doi:10.1002/advs.202410125)
Supplement: Supplementary file 1 — Supporting Information [file ADVS-12-2410125-s001.docx]

Supporting Information

High Quality–Factor All–Dielectric Metacavity for Label–free Biosensing

**Authors**

Yuqiao Zheng^1,2, +^, Jiacheng Sun^3, +^, Yaqing Ma^4^, Hongyong Zhang^2^, Zhen Cui^5^, Giannis G. Paschos^5^, Xixi Song^2^, Ying Tao^2^, Pavlos Savvidis^5^, Wei Kong^4^, Liaoyong Wen^3,^ **^*^**, Sumin Bian^2,^ **^*^** and Mohamad Sawan^2,^ **^*^**.

**Affiliations**

^1^ Zhejiang University, Hangzhou, 310058, Zhejiang, China

^2^ CenBRAIN Neurotech Center of Excellence, School of Engineering, Westlake University, Hangzhou, Zhejiang, 310030, China

^3^ Key Laboratory of 3D Micro/Nano Fabrication and Characterization of Zhejiang, School of Engineering, Westlake University, Hangzhou, Zhejiang, 310030, China

^4^ Advanced Solid-state Semiconductor Lab, School of Engineering, Westlake University, Hangzhou 310024, Zhejiang Province, China

^5^ Key Laboratory for Quantum Materials of Zhejiang Province, Department of Physics, School of Science, Westlake University, Hangzhou, Zhejiang, 310030, China.

^+^ These authors contributed equally to this work

* Corresponding authors. Email: wenliaoyong@westlake.edu.cn; biansumin@westlake.edu.cn; sawan@westlake.edu.cn.

**This PDF file includes:**

Supplementary Text

Figures S1 to S8

Tables S1 to S4

References [1 to 27]

1. Results

1.1. Metacavity design and optimization

We investigated the variation of quality-factor (Q-factor), electric field magnitude and spectra as a function of key parameters: the cavity length (L_cav_), metaatom diameter (d), metaatom height (h) and inter-metaatom distance (a), as illustrated in **Figure S1A** and B respectively. When the bare microcavity resonant at a similar wavelength with the metacavity, the Q-factor of the metacavity is obviously higher than the bare microcavity. The surface sensitivity is investigated by adding an adsorbate layer (0 – 120 nm) at the inner surface of both sensors. As a result, the metacavities consistently exhibit higher surface sensitivity factor (*m*) as depicted in **Figure S2**.

The optical properties of the High-Q factor metacavity (L_cav_ = 3200 nm, a = 400nm, h = 180 nm d = 220 nm) with high near-field EF are presented in **Figure S3A** to D. Figure S3A presents the simulated transmittance spectra of the metacavity, with the electric field distribution of the three modes presented in Figure S3B to D. As a result, due to the lower R-factor of the 2^nd^ mode, both the S*_Bulk_* and *m* factor of the 2^nd^ mode are diminished as illustrated in **Figure S4**.


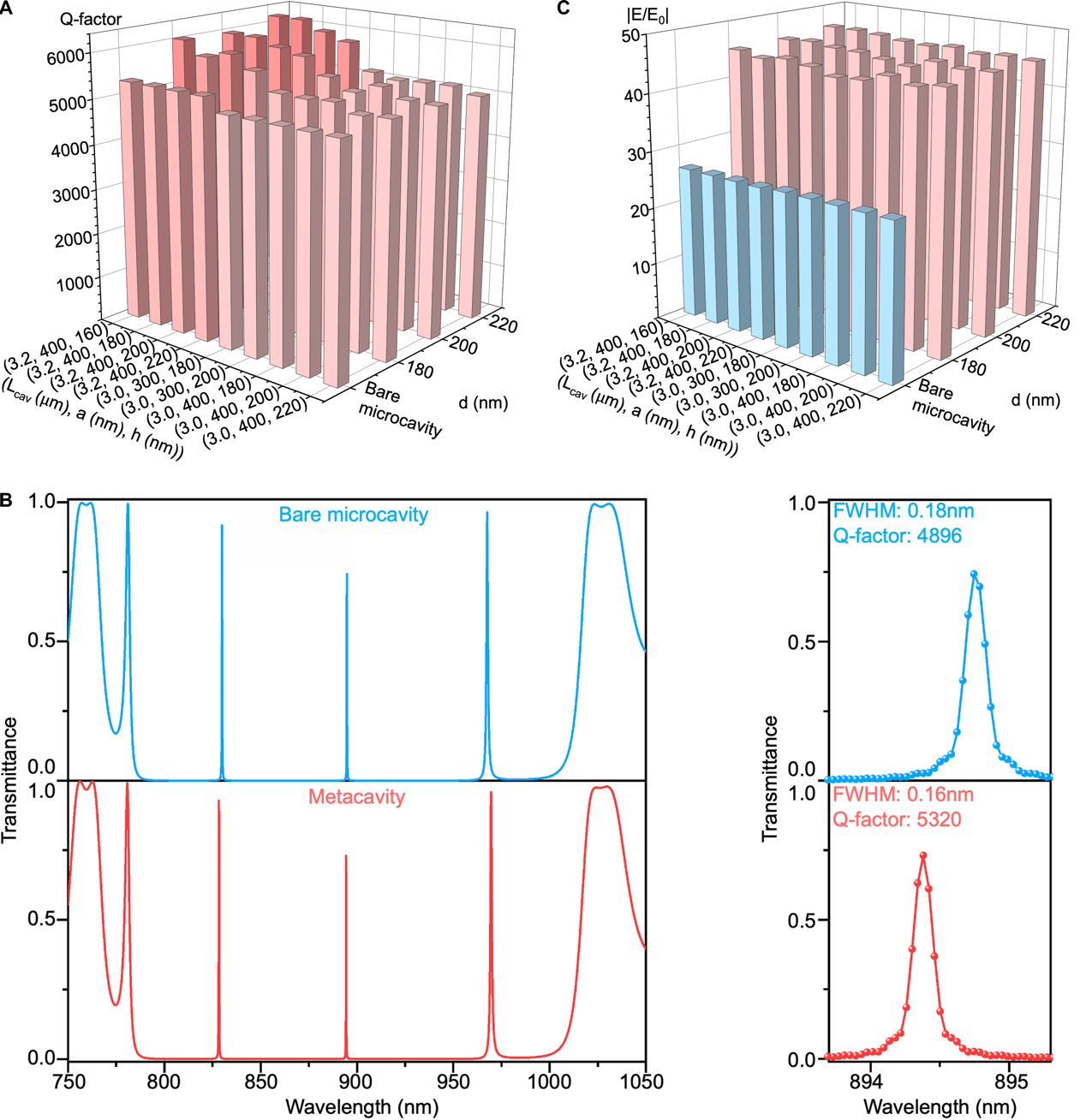


Figure S1. Simulated optical properties of the microcavity sensors. (A) Simulated Q-factor variation of the microcavity sensors with structural parameters (L_cav_, a, h, and d). (B) Comparison of the metacavity (L_cav_ = 3000 nm, a = 400nm, h = 180 nm, d = 200 nm) with the bare microcavity (L_cav_ = 3030 nm). The resonance wavelengths are comparable for the two cavities at the 2^nd^ mode. Left: whole spectra; Right: inset of the 2nd mode. (C) Simulated near field EF variation of the microcavity sensors with structural parameters (L_cav_, a, h, and d).


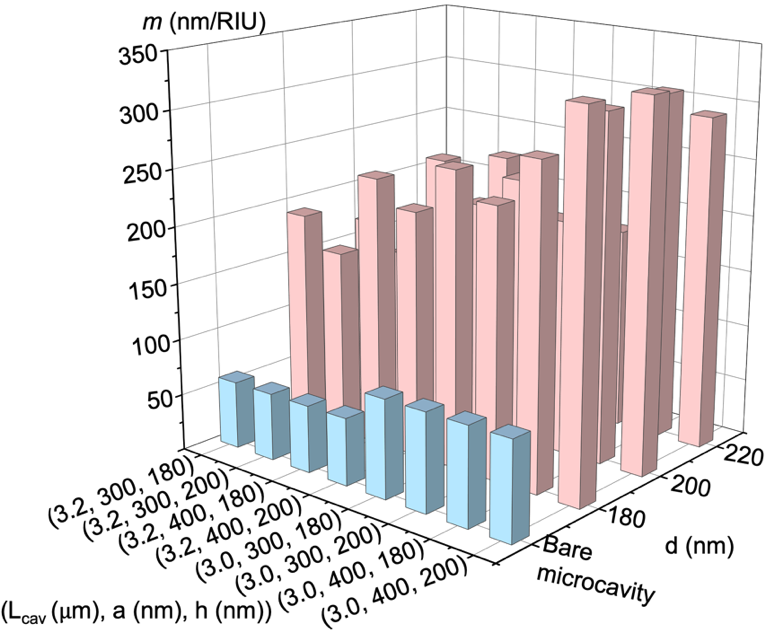


Figure S2. Simulated surface sensitivity factor variation of the microcavity sensors with structural parameters (L_cav_, a, h, and d).


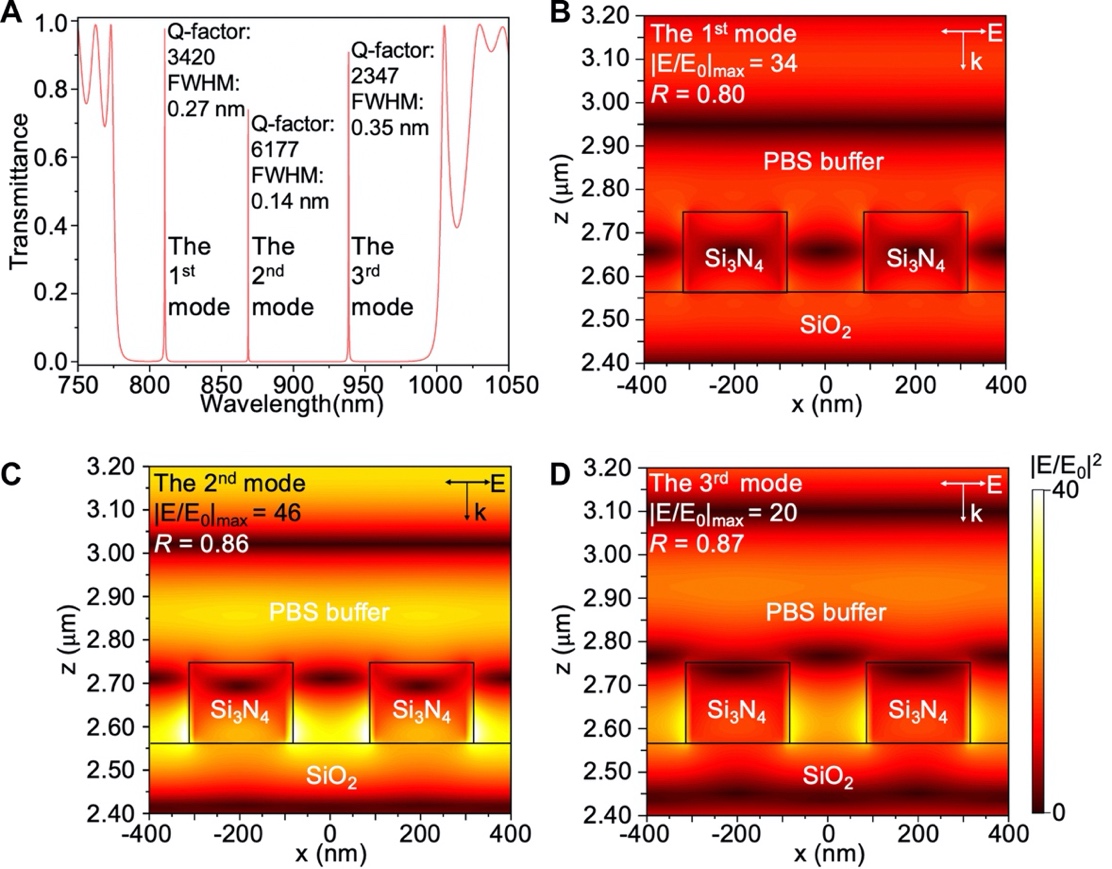


Figure S3. Optical properties of high-Q-factor metacavity (L_cav_ = 3200 nm, a = 400nm, h = 180 nm, d = 220 nm). (A) Transmittance spectrum of the high Q-factor metacavity (L_cav_ = 3200 nm, a = 400nm, h = 180 nm, d = 220 nm). Electric field distribution of the (B) 1^st^, (C) 2^nd^ and (D) 3^rd^ mode of the high-Q-factor metacavity.


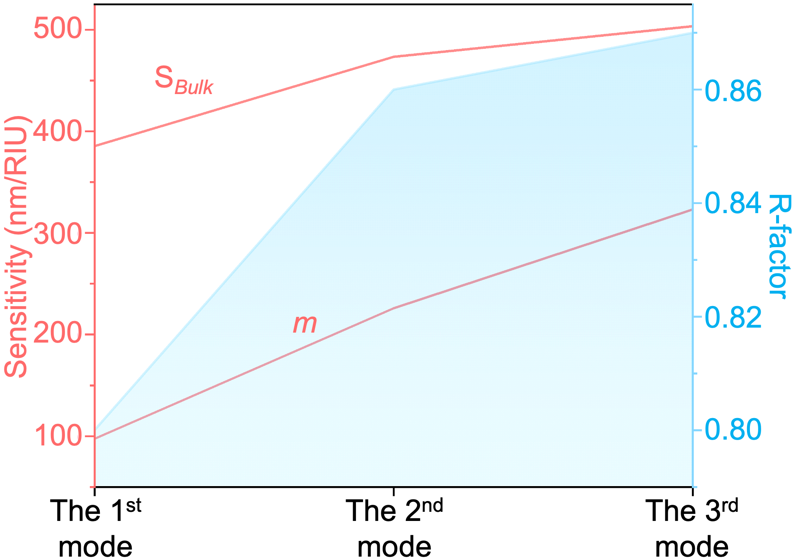


**Figure S4.** The R-factor, S*_Bulk_* and *m* factor of the 1^st^, 2^nd^ and 3^rd^ mode of the high Q-factor metacavity (L_cav_ = 3200 nm, a = 400nm, h = 180 nm, d = 220 nm).

**1.2. Metacavity sensor fabrication and characterization**

After distributed Bragg reflector (DBR) deposition on sapphire wafer, Cu tags with a thickness of 1.45 $\mu m$ were deposited at both edges of the DBR chips. The microcavity was then formed by heat pressing under a consistent pressure of 220 kg at 350 $℃$ for 30 min in a vacuum chamber (**Figure S5A**), thus new Cu-Cu metallic bonding was created between the two separate DBR chips. The middle region between the two DBRs is a 3.0 $\mu m$ hollow cavity, which also serve as a part of the microfluidic channel. Here a real photo of our sensor with microfluidic channel presented in Figure S5B. Figure S5C presents the energy dispersive spectroscopy of the metasurface functionalized DBR, together with the weight percentage of each atom. The cutting process damaged the nanocylinders at the periphery. The experimental Q-factor (Left) and full width at half maximum (FWHM) (Right) of metacavity and bare microcavity devices are shown in Figure S5D.


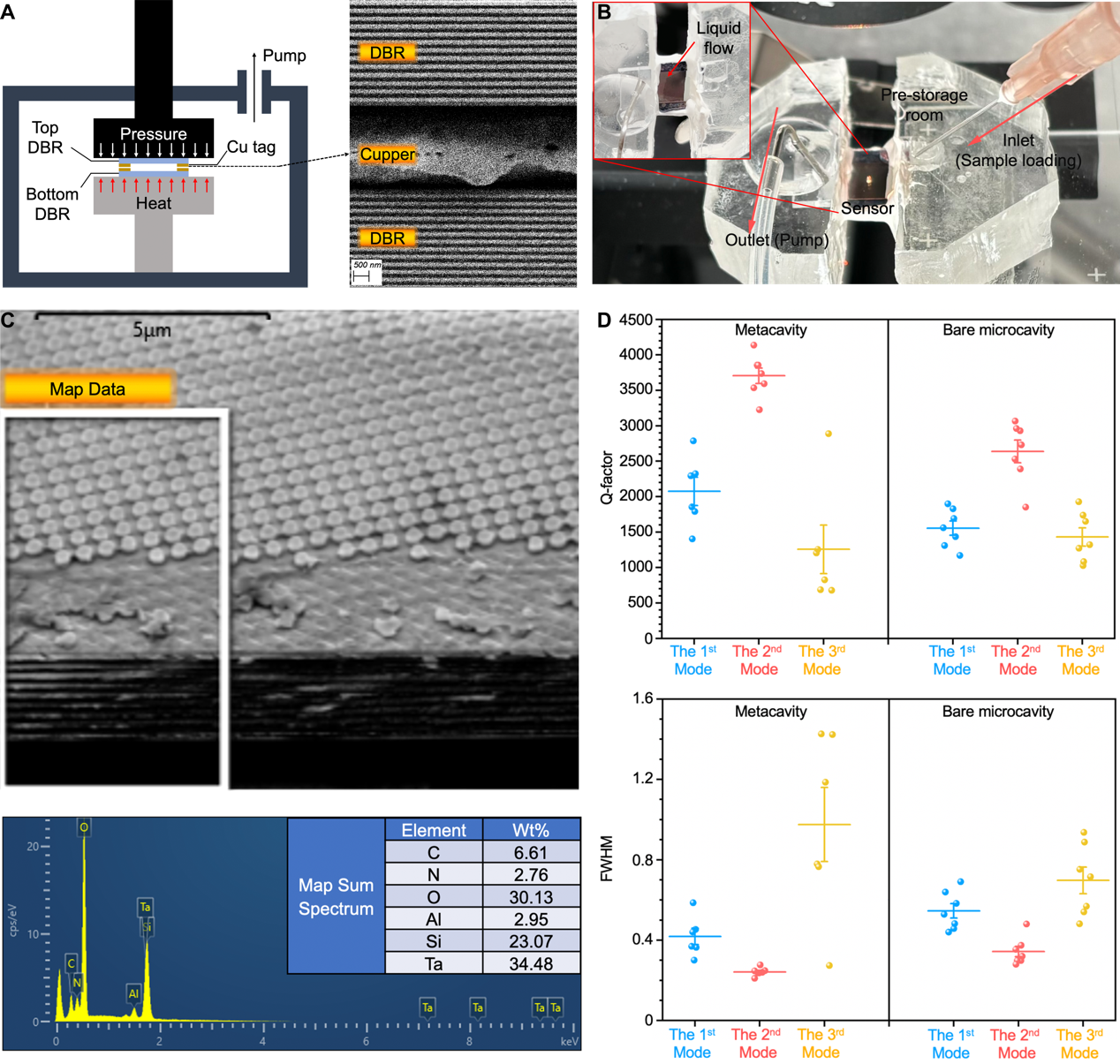


Figure S5. Microcavity formation and characterization. (A) Right: Two separate DBR chips with Cu tags on both edges were bound in vacuum chamber, thus a hollow cavity was formed in the middle as microfluidic channel for sample injection. Left: Cross section SEM image of the formed microcavity. (B) Optical image of the microfluidic system. The sample solution is first injected into a pre-storage room, flowing through the microcavity, and is finally expelled through the outlet by pump. An enlarged view in the upper-left corner details the liquid's filling process within the cavity. (C) Energy dispersive spectroscopy of the metasurface functionalized DBR. (D) Experimental Q-factor (top) and FWHM (bottom) variation of different bare microcavity and metacavity samples.

**1.3. Sensing capacity of the microcavity sensors**

Experimentally, we evaluate the refractometric sensitivity using aqueous ethanol with varying percentages of ethanol, with the transmittance spectra of one of the samples illustrated in **Figure S6A**. The RIs of the solutions are calculated according to the formula provided by A. Shehadeh et al. ^[1]^ and are presented in **Table S1**. Above the RI range of typical bioanalytes, the metacavity exhibits a broad RI linear detection range (Figure S6B), which is a result of its large free spectral range. Comparative analysis with recent nanophotonic resonator sensors in bulk refractometric sensing reveals that the metacavity sensor consistently outperforms them, as summarized in **Table S2**.


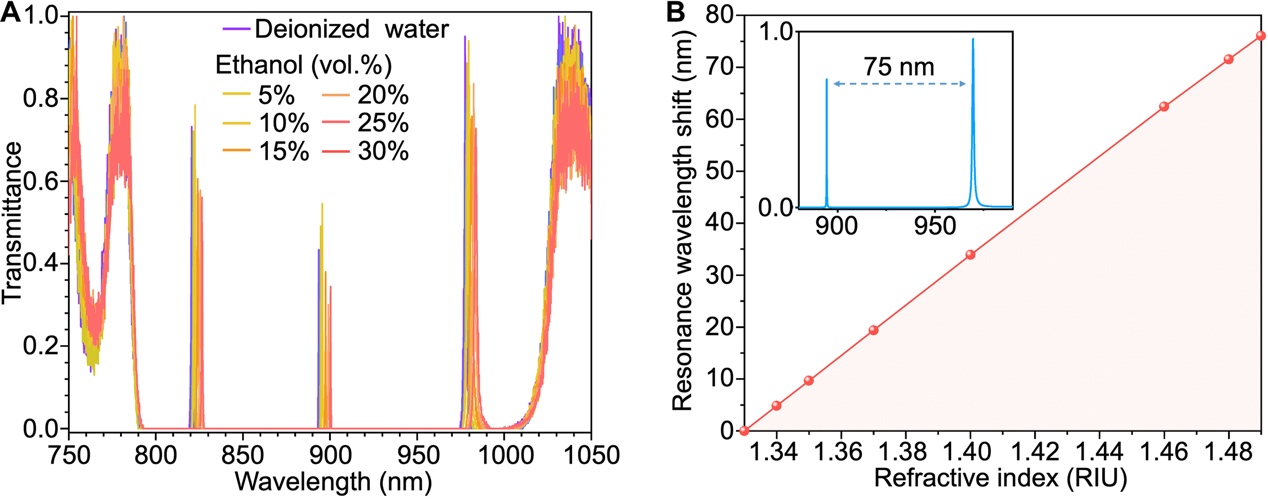


**Figure S6.** Bulk refractometric sensing performance of the metacavity. (A) Experimental normalized transmittance spectra shift of the metacavity with respected to the ethanol concentration change. (B) Simulation resonance wavelength shift of the 2^nd^ mode of the metacavity with respected to RI change above 1.33.

**Table S1.** The RI changes with the volume concentration of ethanol in deionized water.

| Volume ratio of ethanol in deionized water [vol. %] | RI (*n*) |
| --- | --- |
| 0 | 1.3323 |
| 5 | 1.3350 |
| 10 | 1.3376 |
| 15 | 1.3403 |
| 20 | 1.3430 |
| 25 | 1.3457 |
| 30 | 1.3483 |

**Table S2.** Experimental capabilities of recent photonic resonator refractometric sensors.

| Sensor configuration | Experiment RI range [RIU] | FWHM [nm] | Q-factor | S*_Bulk_* [nm/RIU] |
| --- | --- | --- | --- | --- |
| Plasmonic F-P^[2]^ | 1.33 – 1.42 | 2 | 350 | 681 |
| Plasmonic SLR^[3]^ | NA | 0.66 | 2340 | NA |
| SPR^[4]^ | 1.32 – 1.52 | 11 | ~ 77 | 623.7 |
| SPR^[5]^ | 1.3657 – 1.3557 | ~ 50 | ~ 10 | 1198 |
| LSPR^[6]^ | 1.30 – 1.38 | 59 | 9.7 | 435.1 |
| Plasmonic hyperbolic metamaterial^[7]^ | 1.3330 – 1.3336 | ~ 20 | ~ 100 | 30000 |
| Plasmonic SLR^[8]^ | 1.333 – 1.368 | 20 – 25 | ~ 40 | 401 |
| Plasmonic BIC^[9]^ | 1.33 – 1.36 | 6 | 145 | 657 |
| Plasmonic-dielectric BIC^[10]^ | 1.33 – 1.36 | 1.85 | 412 | 492.70 |
| Plasmonic anapole metamaterial^[11]^ | 1.30 – 1.39 | 295 | ~ 5 | 445 |
| Plasmonic Fano resonance ^[12]^ | 1.3418 – 1.3684 | ~ 4 | 200 | 717 |
| All-dielectric Fano resonance^[13]^ | 1.40 – 1.44 | ~ 2 | 483 | 289 |
| All-dielectric Fano resonance^[14]^ | 1.45 – 1.65 | ~ 35 | ~ 40 | 428 |
| All-dielectric BIC^[15]^ | $\Delta RI$ = 0.14 | ~ 5 | 144 | 263 |
| All-dielectric BIC^[16]^ | 1.332 – 1.355 | N/A | 1233 | 301 |
| All-dielectric BIC^[17]^ | 1.333 – 1.380 | ~ 5 | Max. 118.4 | 326 |
| All-dielectric BIC^[18]^ | 1.33441 – 1.33731 | 2.3 | 250 | 305 |
| **This work**  All-dielectric metacavity | 1.332 – 1.348 | Avg. < 0.30  Min. 0.21 | Avg. 3706  Max. 4140 | 449.43 |

F-P: Fabry–Pérot; SPR: surface plasmon resonance; LSPR: localized surface plasmon resonance; BIC: bound stated in continuum.

**1.4. Biosensing capacity of the microcavity sensors by SARS-CoV-2 pseudovirus**

We assessed the efficiency of the proposed silica-binding protein with a core functional domain (cSP) functionalization and the oriented binding of monoclonal antibody (mAb). The process is outlined in **Figure S7A** and includes: (i) and (ii) sensor surface functionalization with cSP, (iii) sensor washing followed by spectrum recording to obtain spectra and response data for cSP functionalization; (iv) mAb loading; (v) subsequent sensor washing and spectrum recording to acquire spectra and response data for mAb binding. Figure S7B displays the normalized transmittance spectra of one metacavity sample at steps (i), (iii), and (vi), while the resonance wavelength shifts of five independent metacavity samples are presented in Figure S7C. The simulation (line) and experimental (error bar chart) Q-factor change with respect to viral load change is presented in Figure S7D.


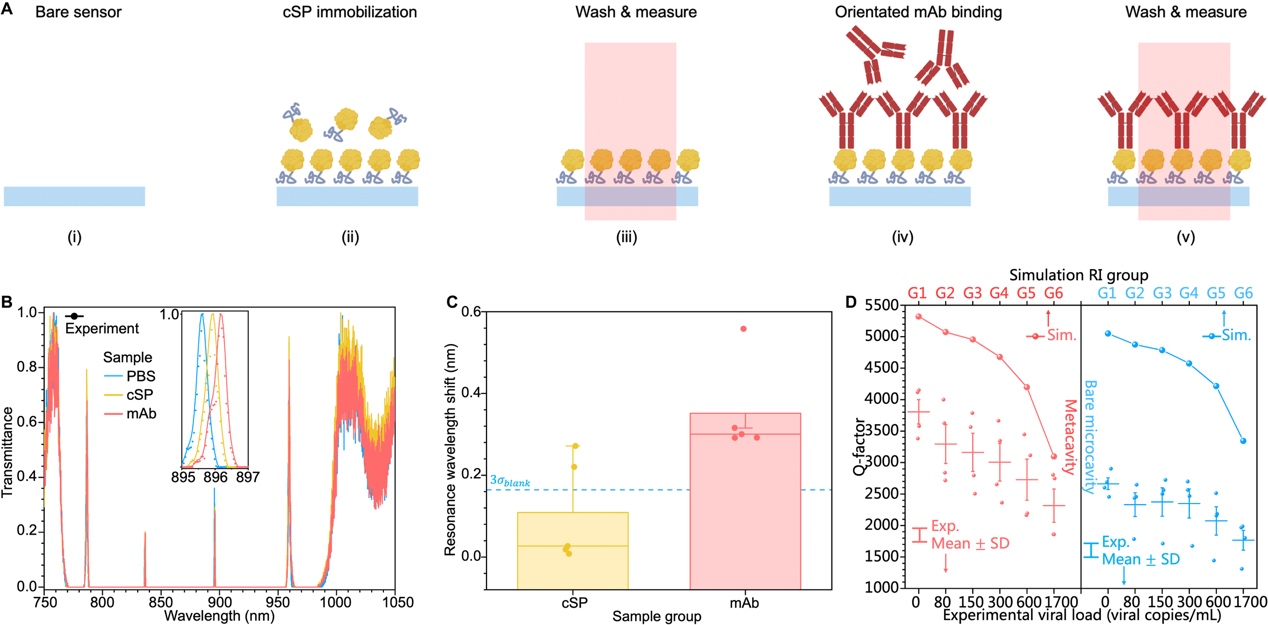


**Figure S7.** Binding efficiency of cSP and mAb. (A) Process flow in biofunctionalization validation. (i) and (ii) cSP protein binding on the bare sensor surface; (iii) Sensor washing and spectra measurement; (iv) Orientated functionalization of mAb on the sensing surface; (v) Sensor washing and spectra measurement. (B) One of the metacavity sample with PBS filling the cavity (blue), cSP binding on the surface (yellow) and orientated mAb binsing on the surface (red). (C) Resonance wavelength shift with respected to cSP immobilization (yellow) and mAb binding (red). The blue dashed line indicates the standard deviation of the bare sensor. (D) Simulated Q-factor changes in the metacavity (red line) and bare microcavity (blue line) sensors in response to RI (n, k) change. Experimental Q-factor changes in the metacavity (red error bar chart) and bare microcavity (blue error bar chart) sensors in response to different virus concentrations.

**2. Methods**

We measured the RIs of key materials and reagents used in this work by ellipsometer, including the RI(*n*,*k*) of Ta_2_O_5_,(**Figure S8A**) and the RI (*n*) of sapphire wafer (Figure S8B) and PBS buffer (1$\times$) (Figure S8C). The surface sensitivity for detecting surface-bound viral particles was evaluated through simulation, using the measured refractive index (*n*, *k*) of the viral solution (Figure 1B and Figure S8D). We compiled a set of (*n*, *k*) values for the surface sensitivity calculation, as detailed in **Table S3**. We compared the recent studies in SARS-CoV-2 whole virus detection as presented in **Table S4**. As a result, the metacavity exhibits competitive LoD across various biosensing platforms.


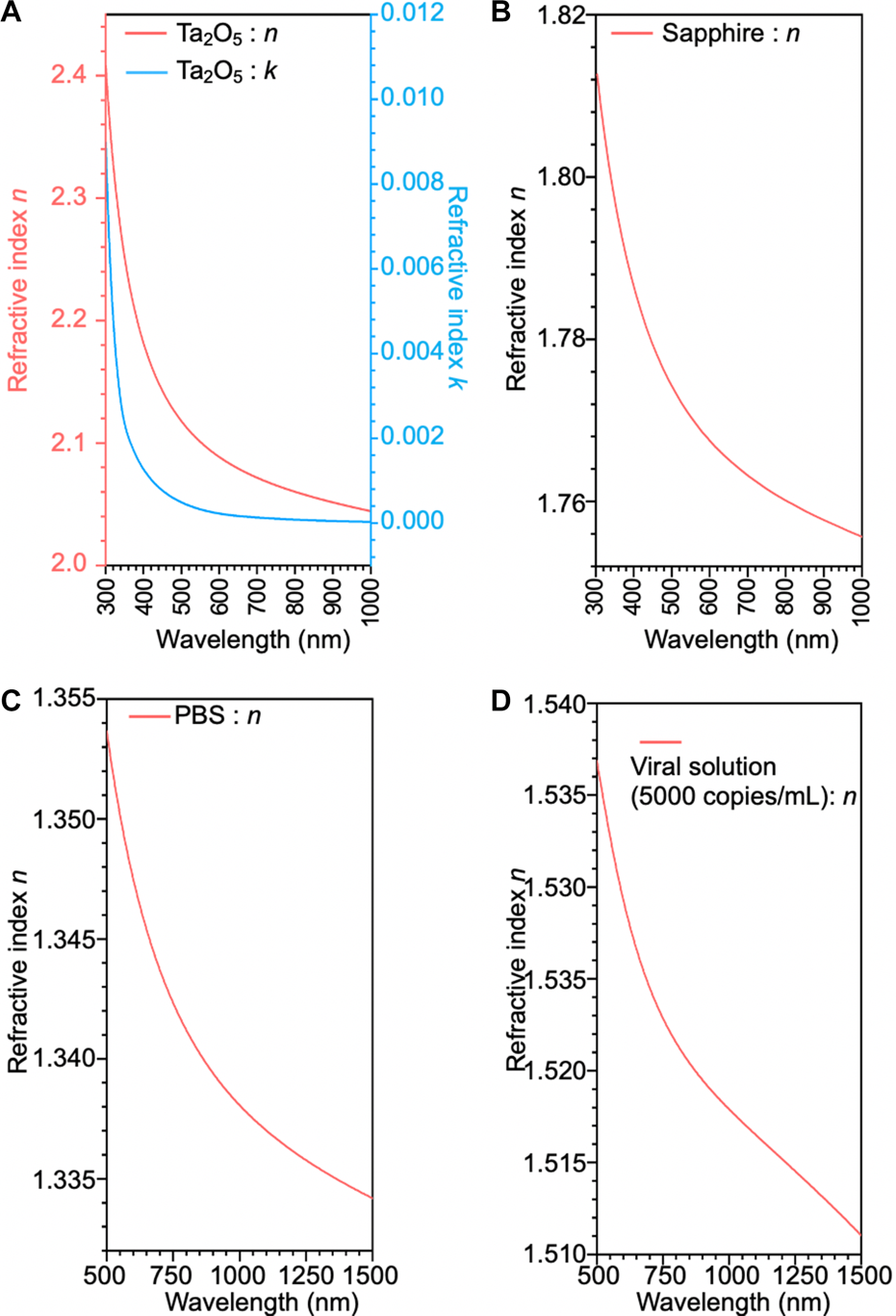


**Figure S8.** Experimental RI properties of the key materials and reagents in this work. (A) The measured RI (*n,k*) of the Ta_2_O_5_. The measured RI (*n*) of (B) sapphire wafer, (C) PBS buffer (1$\times$) and (D) SARS-CoV-2 pseudovirus solution with viral concentration of 5000 copies/mL.

**Table S3.** Simulation RI (*n*, *k*) values for metacavity biosensing performance evaluation.

| RI group | RI (*n*) | RI(*k*) [$\times$10^-6^] |
| --- | --- | --- |
| Group 1 (G1) | 1.34000 | 0.00 |
| Group 2 (G2) | 1.34288 | 31.12 |
| Group 3 (G3) | 1.34540 | 58.35 |
| Group 4 (G6) | 1.35080 | 116.70 |
| Group 5 (G5) | 1.36160 | 233.40 |
| Group 6 (G6) | 1.40120 | 661.30 |

**Table S4.** Performance of biosensing platforms in SARS-CoV-2 whole virus detection.

| Configuration | Target | LoD [copies/mL] |
| --- | --- | --- |
| FET ^[19]^ | Synthetic virus | 165 |
| FET ^[20]^ | Nasopharyngeal Swab | 242 |
| Voltammetric ^[21]^ | Nasal swab | 2559 |
| Voltammetric ^[22]^ | Saliva | 20 |
| Voltammetric ^[23]^ | Saliva | 1000 |
| SERS ^[24]^ | Inactivated virus | 80 |
| SERS ^[25]^ | Nasal and throat swabs | 100 |
| SPR ^[26]^ | Pseudovirus mixed with nasal mucosa | 1725 |
| Lens-free holographic microscope ^[27]^ | Pseudovirus | 1270 |
| Metacavity ^[This work]^ | Pseudovirus | 119 |

FET: field-effect transistor; SERS: surface enhanced Raman scattering; SPR: surface plasmon resonance.

**References**

[1] A. Shehadeh, A. Evangelou, D. Kechagia, P. Tataridis, A. Chatzilazarou, F. Shehadeh, *Food Chem.* **2020**, *329*, 127085.

[2] Y. Shen, K. He, Q. S. Zou, S. Xing, J. Y. Huang, M. C. Zheng, X. Y. She, C. J. Jin, *Adv. Funct. Mater.* **2022**, *32*, 2108741.

[3] M. S. Bin-Alam, O. Reshef, Y. Mamchur, M. Z. Alam, G. Carlow, J. Upham, B. T. Sullivan, J. M. Ménard, M. J. Huttunen, R. W. Boyd, K. Dolgaleva, *Nat Commun.* **2021**, *12*, 974.

[4] S. S. Ye, X. M. Zhang, L. X. Chang, T. Q. Wang, Z. B. Li, J. H. Zhang, B. Yang, *Adv. Optical Mater.* **2014**, *2*, 779-787.

[5] C. Zhang, Z. Li, S. Z. Jiang, C. H. Li, S. C. Xu, J. Yu, Z. Li, M. H. Wang, A. H. Liu, B. Y. Man, *Sens. Actuators B: Chem.* **2017**, *251*, 127-133.

[6] J. X. Cai, C. P. Zhang, C. W. Liang, S. Y. Min, X. Cheng, W. D. Li, *Adv. Optical Mater.* **2019**, *7*, 1900516.

[7] K. V. Sreekanth, Y. Alapan, M. ElKabbash, E. Ilker, M. Hinczewski, U. A. Gurkan, A. De Luca, G. Strangi, *Nat. Mater.* **2016**, *15*, 621.

[8] A. Danilov, G. Tselikov, F. Wu, V. G. Kravets, I. Ozerov, F. Bedu, A. N. Grigorenko, A. V. Kabashin, *Biosens. Bioelectron.* **2018**, *104*, 102-112.

[9] Y. Zhou, Z. H. Guo, X. Y. Zhao, F. L. Wang, Z. Y. Yu, Y. Z. Chen, Z. R. Liu, S. Y. Zhang, S. L. Sun, X. Wu, *Adv. Optical Mater.* **2022**, *10*, 2200965.

[10] M. Luo, Y. Zhou, X. Y. Zhao, Z. H. Guo, Y. X. Li, Q. Wang, J. J. Liu, W. Luo, Y. Z. Shi, A. Q. Liu, X. Wu, *Acs Nano* **2024**, *18*, 6477-6486.

[11] J. Yao, J. Y. Ou, V. Savinov, M. K. Chen, H. Y. Kuo, N. I. Zheludev, D. P. Tsai, *Photonix* **2022**, *3*, 23.

[12] A. A. Yanik, A. E. Cetin, M. Huang, A. Artar, S. H. Mousavi, A. Khanikaev, J. H. Connor, G. Shvets, H. Altug, *Proc. Natl. Acad. Sci. USA* **2011**, *108*, 11784-11789.

[13] Y. M. Yang, Kravchenko, II, D. P. Briggs, J. Valentine, *Nat Commun.* **2014**, *5*, 5753.

[14] K. E. Chong, H. Orton, I. Staude, M. Decker, A. E. Miroshnichenko, I. Brener, Y. S. Kivshar, D. N. Neshev, *Philos. Trans. R. Soc. A* **2017**, *375*, 20160070.

[15] F. Yesilkoy, E. R. Arvelo, Y. Jahani, M. K. Liu, A. Tittl, V. Cevher, Y. Kivshar, H. Altug, *Nat. Photonics* **2019**, *13*, 390.

[16] K. Watanabe, M. Iwanaga, *Nanophotonics* **2023**, *12*, 99-109.

[17] J. Wang, J. Kühne, T. Karamanos, C. Rockstuhl, S. A. Maier, A. Tittl, *Adv. Funct. Mater.* **2021**, *31*, 2104652.

[18] Y. Jahani, E. R. Arvelo, F. Yesilkoy, K. Koshelev, C. Cianciaruso, M. De Palma, Y. Kivshar, H. Altug, *Nat Commun.* **2021**, *12*, 3246.

[19] S. Park, H. Kim, K. Woo, J. M. Kim, H. J. Jo, Y. Jeong, K. H. Lee, *Nano Lett.* **2022**, *22*, 50-57.

[20] G. Seo, G. Lee, M. J. Kim, S. H. Baek, M. Choi, K. B. Ku, C. S. Lee, S. Jun, D. Park, H. G. Kim, S. J. Kim, J. O. Lee, B. T. Kim, E. C. Park, S. I. Kim, *Acs Nano* **2020**, *14*, 5135-5142.

[21] A. Alam, T. Uppal, M. S. Islam, M. Misra, S. C. Verma, *ACS Omega* **2023**, *8*, 45700-45707.

[22] H. Zargartalebi, H. Yousefi, C. D. Flynn, S. Gomis, J. Das, T. L. Young, E. Chien, S. Mubareka, A. McGeer, H. S. Wang, E. H. Sargent, A. S. Nezhad, S. O. Kelley, *J. Am. Chem. Soc.* **2022**.

[23] B. M. Szydlowska, C. C. Pola, Z. Z. Cai, L. E. Chaney, J. A. Hui, R. Sheets, J. Carpenter, D. Dean, J. C. Claussen, C. L. Gomes, M. C. Hersam, *ACS Appl. Mater. Interfaces* **2024**, *16*, 25169-25180.

[24] Y. Yang, Y. S. Peng, C. L. Lin, L. Long, J. Y. Hu, J. He, H. Zeng, Z. R. Huang, Z. Y. Li, M. Tanemura, J. L. Shi, J. R. Lombardi, X. Y. Luo, *NANO-MICRO LETT* **2021**, *13*, 109.

[25] Y. Y. Li, C. L. Lin, Y. S. Peng, J. He, Y. Yang, *Sens. Actuators B: Chem.* **2022**, *365*, 131974.

[26] C. Y. Lin, W. H. Wang, M. C. Li, Y. T. Lin, Z. S. Yang, A. N. Urbina, W. Assavalapsakul, A. Thitithanyanont, K. R. Chen, C. C. Kuo, Y. X. Lin, H. H. Hsiao, K. D. Lin, S. Y. Lin, Y. H. Chen, M. L. Yu, L. C. Su, S. F. Wang, *Bioengineering & Translational Medicine* **2023**, *8*, e10410.

[27] C. J. Potter, Y. M. Hu, Z. Xiong, J. Wang, E. McLeod, *Lab Chip* **2022**, *22*, 3744-3754.
